# Case of Extensive Ulceration of the Soft Palate and Tonsils

**Published:** 1824-05

**Authors:** J. Bacot

**Affiliations:** Surgeon to the St. George's and St. James's Dispensary.


Art. VI.—*Case of extensive Ulceration of the Soft Palate and Tonsils.* By J. Bacot, Surgeon to the St. George's and St. James's Dispensary.

Among the almost infinite variety of symptoms usually ascribed to the action of the venereal virus, there is no one more frequently met with than an ulcerated state of the soft palate and tonsils; and so completely has the connexion between lues venerea and ulceration of these parts been established, in the opinion of many practitioners, that mercury is not uncommonly had recourse to, without pause or hesitation, whenever a breach of surface, to any extent, is detected in those parts; and very often without any reference to the previous history, or accompanying circumstances, of the case. The result of such practice is almost invariably a rapid extension of the malady, which quickly spreads to the periosteum, and finally to the soft and tender bones of the palate and nose; exfoliation ensues; and that fatal destruction of the parts, which is indeed the consequence of an inconsiderate and rash introduction of mercury into the system, is attributed either to the especial malignity of the disease, or to the remedy having either been delayed too long or not pushed to a sufficient extent. It is true that nature, in these cases, finally triumphs over both the disease and the remedy; but the destruction of bone cannot be supplied, and a serious defect, or deformity, is the necessary result. It will not, I trust, be suspected that I am an advocate for the cure, or rather the attempt to cure, *all* ulcers of the tonsils or palate without the aid of mercury: on the contrary, I am fully convinced that there is no surer, or more judicious, mode of treatment in all those cases that are really syphilitic, provided there be nothing in the constitution forbidding the exhibition of that medicine. But, in proportion to the extent of my confidence in the powers of mercury, so also is my desire to discriminate certain affections of the throat, bearing some resemblance to the disease above mentioned, but in which mercury is absolutely hurtful, and attended with the most serious consequences; and to draw the attention of the young practitioner especially to an ulcerated condition of the soft palate and tonsils, which I have frequently met with, more particularly among the poorer classes of society: the subjects of this affection being generally those of a decidedly strumous habit, with constitutions broken down by previous disease, and where, as far as I have been able to learn, there has been no cause to suspect the disease to have arisen from any primary ulcerations of the genital organs; though, even had I been able to trace such an origin, I should still maintain the inadmissibility of mercury in these cases.

In making these preliminary observations, I beg to remark, that I have no hypothesis to offer or to defend: I am merely stating plain facts; and I select the following case (from many others) as illustrative of my meaning, being not only one of the strongest, as far as the long continuance, inveteracy, and extent, of the disease is concerned, but also as one of the most recent which has fallen within my notice. I may also add, that it is well known to, and was seen in its progress several times, by my friend and colleague, Dr. Gregory.

Sarah Faulkner, widow, aged forty years, came under my care at the St. George's and St. James's Dispensary, on the 16th day of January. She had been suffering for upwards of two months with an ulcerated throat, attended with difficulty in swallowing, great emaciation, and general disturbance of the system. When I first saw and examined her throat, I found an extensive sloughy surface occupying the whole of the velum pendulum palate, and including both the tonsils, with a thickened margin of a deep-red colour. There was a profuse sanious discharge through the nostrils, attended with an intolerable stench; a blush of inflammation extended across the ridge of the nose; great pain was experienced in every attempt to swallow fluids; the pulse was 110, the skin hot, and a sense of restlessness and general uneasiness prevented sleep. It had been suspected that these symptoms proceeded from some primary venereal infection, but this the patient declared to be impossible. Considering the great emaciation of this woman, and the length of time which the disease had continued, I did not think myself justified in bleeding generally from the system, but six leeches were twice applied to the external fauces, with an interval of three days between each application. A brisk purgative was administered, and antimony given, in the form of a mixture containing a drachm of the liquor antimonii tartarizati to the half-pint, so as to produce the effect of nausea and slight sickness, which was kept up during the greater part of twenty-four hours.

It is not necessary to give a minute daily detail of the progress of the case under this mode of treatment; but the alteration in the character of the sore, as soon as the nauseating effects of the antimony became apparent, were remarkable: the elevated and inflamed edges of the ulcers disappeared, a rapid exfoliation of the sloughs took place, and on the 20th March the ulcers were completely cicatrised, leaving scarcely a trace of the formidable disease that had existed only a fortnight previously. During the whole of this time the strictest antiphlogistic regimen was maintained: nothing more than gruel, or milk and water, was permitted to be taken; and no local application was made use of to the part, excepting a gargle of the acetate of lead diluted with water, in the first instance, and afterwards a solution of mel eruginis applied upon a piece of rag to the sloughy surfaces.

On the 29th March, the patient was discharged cured, having rapidly recovered her flesh and strength; the inflammatory blush upon the nose had entirely disappeared, as well as the discharge from the nostrils.

A very few words of comment are all that I shall consider it necessary at present to subjoin to the detail of this case. The particular circumstances which I conceive forbid the use of mercury in affections of the palate and tonsils, are, first, the deep-coloured inflammation surrounding them, and, if mercury is intended to be exhibited, it cannot be safely done until this condition of the sores is removed; 2dly, the *pain* in swallowing is not a mere *difficulty* arising from the fauces being narrowed by the enlargement of the tonsils; it is actually a vivid pain, occurring more especially when any thing, however mild, whether it be fluid or solid, touches the diseased parts; 3dly, the heat of skin and quickened state of the circulation; and, 4thly, the restlessness and feverish exacerbation at night. In the case I have recorded, all these circumstances were strongly marked; and it was not until much blood had been lost locally, and the system was brought fully under the nauseating effect of antimony, that the striking and rapid alteration in the state of the throat took place.

The above four circumstances sufficiently distinguish this affection of the throat from the common syphilitic ulcer of the tonsils or palate, in which there is scarcely any surrounding inflammation, and but little sensibility. The general health and appetite are not much, if at all, impaired, although there is often a considerable wasting of the flesh. The pulse is not affected, and the sleep is undisturbed.

I have had occasion to see cases similar to that of Sarah Faulkner occurring in the young and sanguine; in those especially who have lived very freely, particularly as far as regards the abuse of ardent spirits; and in those instances I have found it necessary to adopt a free use of the lancet prior to the exhibition of the tartarised antimony; the blood drawn exhibiting the most decided appearances of inflammation. When the progress of the ulceration is stopped, and not until then, I commence the administration of the compound decoction of sarsaparilla, to the extent of a pint or a pint and a half in the course of the day; under the use of which recovery is in general most rapid.

---

